# The US Anti-Prostitution Pledge: A Call for Cooperation

**DOI:** 10.1371/journal.pmed.0040280

**Published:** 2007-10-30

**Authors:** Jay Silverman, Michele Decker

Masenior and Beyrer's article is an important contribution to the continuing debate as to the direction of public health efforts regarding commercial sex work [1]. The authors are correct in that the debate between those focused on the social and economic rights of prostituted women and girls as “workers” and those focused on preventing the trafficking of women and girls into prostitution has resulted in precious little dialogue on how to best proceed.

Regrettably, as illustrated by the authors, the US anti-prostitution pledge may well have contributed to the polarization of these two groups. However, to make progress, multiple realities must be acknowledged and considered. The authors present the conflation of sex trafficking and prostitution as a barrier to effective action and policy that must be eliminated; however, compelling evidence exists to support such conflation.

Specifically, approximately half of sex workers are prostituted as minors [2,3]. Studies including prostituted minors indicate that virtually all were trafficked into sex work [4], and studies of sex workers across multiple countries indicate that the majority would prefer to leave prostitution if it were safe and economically feasible to do so [2,5]. Thus, anti-trafficking advocates may reasonably contend that maintaining the institution of sex work through decriminalization and organization of health and social welfare programs will likely lead to continued trafficking of women and girls to maintain this highly profitable activity.

On the other hand, as presented by Masenior and Beyrer, the many thousands of women and girls involved in commercial sex work need and deserve assistance based on the tremendous health risks they suffer (e.g., HIV infection). However, those focused on promoting the health of sex workers have often inadequately considered the presence of minor girls [6] or trafficking victims in the sex work venues within which they operate [7]. While such programs appear to empower adults purporting to be engaged in voluntary sex work, no evidence suggests that these efforts contribute to reducing the numbers or protecting the health of trafficked women and girls.

While we cannot reasonably make the assumption that all sex workers are trafficked, we also cannot reasonably accept that all sex workers are voluntarily prostituted. Amazingly, we have yet to conduct adequate research to answer this question. Thus the conflation of sex work and trafficking continues, as lamented by the authors. To move forward, both sides must work together based on their common goal of improving the health and well-being of this highly vulnerable population. Advocates, practitioners, and researchers representing both sides of this debate must dialogue to find effective means of reducing trafficking of women and girls for sexual exploitation, and also to find ways to assist sex workers to minimize the health risks they face, while simultaneously remaining vigilant in detecting and humanely assisting prostituted children and adult trafficking victims.
